# A randomized, double-blind, placebo-controlled study to evaluate the addition of methotrexate to etanercept in patients with moderate to severe plaque psoriasis

**DOI:** 10.1111/j.1365-2133.2012.11015.x

**Published:** 2012-09

**Authors:** AB Gottlieb, RG Langley, BE Strober, KA Papp, P Klekotka, K Creamer, EHZ Thompson, M Hooper, G Kricorian

**Affiliations:** 1 Tufts Medical CenterBoston, MA, U.S.A.; 2 Dalhousie UniversityHalifax, NS, Canada; 3 University of Connecticut School of MedicineFarmington, CT, U.S.A.; 4 Probity Medical ResearchWaterloo, ON, Canada; 5 Amgen Inc.Thousand Oaks, CA, U.S.A.

## Abstract

**Background:**

Etanercept plus methotrexate combination therapy has not been adequately investigated in psoriasis.

**Objectives:**

To evaluate etanercept plus methotrexate vs. etanercept monotherapy in patients with moderate to severe plaque psoriasis who had not failed prior methotrexate or tumour necrosis factor-inhibitor therapy.

**Methods:**

Patients received etanercept 50 mg twice weekly for 12 weeks followed by 50 mg once weekly for 12 weeks and were randomized 1 : 1 to receive methotrexate (7·5–15 mg weekly) or placebo. The primary endpoint was the proportion of patients achieving ≥75% improvement in Psoriasis Area and Severity Index (PASI 75) at week 24.

**Results:**

In total, 239 patients were enrolled in each arm. PASI 75 was significantly higher at week 24 for the combination therapy group compared with the monotherapy group (77·3% vs. 60·3%; *P* < 0·0001). Other PASI improvement scores at week 12 [PASI 75, 70·2% vs. 54·3% (*P* = 0·01); PASI 50, 92·4% vs. 83·8% (*P* = 0·01); and PASI 90, 34·0% vs. 23·1% (*P* = 0·03)] showed similar results as did week 24 PASI 50 (91·6% vs. 84·6%; *P* = 0·01) and PASI 90 (53·8% vs. 34·2%; *P* = 0·01). Significantly more patients receiving combination therapy than monotherapy had static Physician’s Global Assessment of clear/almost clear at week 12 (65·5% vs. 47·0%; *P* = 0·01) and week 24 (71·8% vs. 54·3%; *P* = 0·01). Adverse events (AEs) were reported in 74·9% and 59·8% of combination therapy and monotherapy groups, respectively; three serious AEs were reported in each arm.

**Conclusions:**

Combination therapy with etanercept plus methotrexate had acceptable tolerability and increased efficacy compared with etanercept monotherapy in patients with moderate to severe psoriasis.

Psoriasis is a chronic inflammatory disease with a prevalence of approximately 1–5% in the general population.^1^ The majority of diagnosed patients have plaque psoriasis, and about 20% have moderate to severe disease.^2^ Because of the chronic nature of psoriasis, patients typically need long-term treatment, even though traditional systemic therapies are often associated with cumulative, dose-related toxicities.^3^ Thus, the current focus for treatment of patients with moderate to severe psoriasis is intermittent or combination therapy to overcome loss of efficacy over time and the cumulative toxicities of traditional agents.^4^–^7^

Etanercept is a fully human, soluble tumour necrosis factor (TNF)–receptor fusion protein that is approved to treat moderate to severe plaque psoriasis at a dosage of 50 mg twice weekly for up to 3 months, followed by 50 mg once weekly.^8^,^9^ Etanercept has been shown to improve disease symptoms and health-related quality of life while maintaining an acceptable safety and tolerability profile in patients with moderate to severe plaque psoriasis.^10^–^16^

Methotrexate is a systemic therapy that has demonstrated clinical efficacy in patients with psoriasis. In a randomized controlled trial, methotrexate was significantly more effective at 16 weeks than placebo (36·5% vs. 18·9%; *P* < 0·05) but less effective than adalimumab (79·6%; *P* < 0·001) in reducing the Psoriasis Area and Severity Index (PASI) by 75% (PASI 75) in patients with moderate to severe plaque psoriasis.^17^ Similarly, a recent randomized, controlled study reported that infliximab was significantly more effective than methotrexate at 16 weeks in patients with moderate to severe psoriasis in terms of PASI 75.^18^ Methotrexate has also been found to be effective at inducing and maintaining remission in patients with moderate to severe psoriasis in open-label and retrospective studies and in a small randomized comparative trial.^19^,^20^ Combination therapy with methotrexate plus other systemic treatments (e.g. ciclosporin, acitretin) or phototherapy is an approach that is used to treat patients with psoriasis.^4^,^5^ Despite its efficacy and acceptable tolerability in patients with rheumatoid arthritis (RA),^21^–^25^ the combination of etanercept plus methotrexate has not been widely investigated in psoriasis. One small single-centre case series reported that the combination could be effective in patients with insufficient response to etanercept monotherapy.^26^ More recently, a small study compared etanercept plus either continuous or tapered/discontinued methotrexate in 59 patients with moderate to severe psoriasis.^27^ Disease symptoms were greatly improved at 24 weeks in the combination arm and adverse events (AEs) were similar in both groups.^27^ The objective of this study was to assess the efficacy and safety of the addition of methotrexate to etanercept compared with etanercept monotherapy in patients with moderate to severe plaque psoriasis using a randomized, double-blind, placebo-controlled, multicentre design.

## Patients and methods

### Patients

The independent ethics committee/institutional review board of each participating centre approved the protocol. All patients provided written informed consent before performance of any study-related procedures. The study was conducted in accordance with International Conference on Harmonisation Good Clinical Practice regulations and guidelines. The trial is registered in the U.S. National Institutes of Health clinicaltrials.gov database, identifier NCT01001208.

Eligible patients (aged ≥ 18 years) had stable moderate to severe plaque psoriasis for ≥ 6 months, psoriasis involving ≥ 10% of body surface area (BSA), a PASI ≥ 10 at screening and baseline, and were candidates for systemic therapy or phototherapy in the opinion of the investigator. Adequate haematological (haemoglobin ≥ 11 g dL^−1^, white blood cell count ≥ 3·5 × 10^9^ cells L^−1^, platelet count ≥ 125 × 10^9^ cells L^−1^), renal (creatinine clearance ≥ 50 mL min^−1^) and hepatic (serum total bilirubin < 1·5 mg dL^−1^, serum albumin > 3·5 mg dL^−1^) function was required. Serum glutamic oxaloacetic transaminase/aspartate aminotransferase (SGOT/AST) and glutamic pyruvic transaminase/alanine aminotransferase (SGPT/ALT) levels had to be within normal limits at screening.

Exclusion criteria included active guttate, erythrodermic, or pustular psoriasis or other skin conditions at screening that would interfere with study evaluation; concurrent significant medical conditions; active Common Terminology Criteria (CTC) for Adverse Events version 2.0 grade ≥ 2 infection within 30 days of screening; history of significant methotrexate toxicity or total cumulative methotrexate exposure > 1000 mg (unless grade ≥ IIIb liver injury has not occurred); use of ultraviolet (UV) B therapy, topical ciclosporin or calcineurin inhibitors, class III through VII topical corticosteroids (permitted on the scalp, axillae, and/or groin), or topical vitamin A or D analogues within 14 days of screening; and psoralen or UVA therapy, systemic psoriasis therapy (including methotrexate), oral retinoids, class I or II topical corticosteroids, dithranol, cyclophosphamide, sulfasalazine, or intravenous or oral calcineurin inhibitors within 28 days of screening. Patients were excluded if they had received a TNF-blocking agent or other biologics within 3 months or interleukin (IL)-12 or IL-23 inhibitors within 6 months of study initiation. Prior use of methotrexate or etanercept did not exclude enrollment. However, patients were excluded if they had experienced a clinically significant AE with prior use of methotrexate or experienced lack of efficacy or a clinically significant AE with prior use of a TNF-blocker.

### Study design and treatment

**Fig 1:**
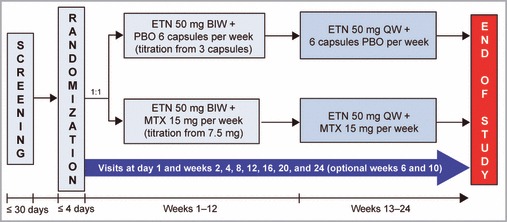
Study schema. BIW, twice weekly; ETN, etanercept; MTX, methotrexate; PBO, placebo; QW, once weekly.

This was a randomized, double-blind, placebo-controlled, multicentre, phase IIIb study. All patients received etanercept 50 mg subcutaneously twice weekly for 12 weeks followed by 50 mg once weekly for an additional 12 weeks and were randomized 1 : 1 to oral methotrexate (combination group) titrated from 7·5 mg (weeks 1–2) to 10 mg (weeks 3–4) to a maximum of 15 mg or the maximum tolerated dose (weeks 5–24) or matching placebo (monotherapy group) (Fig. 1). Depending on patient tolerability, titration could be paused or the final dose could be lower than 15 mg. Randomization was performed within each of the four strata [body mass index (BMI) ≤ 35 vs. > 35 kg m^−2^ and TNF-blocker exposure status] to achieve balance, and enrolment of patients with prior TNF-blocker exposure was to be capped at one half of the planned study population.

### Endpoints and assessments

The primary endpoint was a comparison of the proportion of patients achieving PASI 75 from baseline to week 24. Secondary efficacy endpoints included PASI 75 at week 12, PASI 50 and PASI 90 (PASI improvement from baseline of ≥ 50% and ≥ 90%, respectively) at weeks 12 and 24, static Physician’s Global Assessment (sPGA) of clear/almost clear at weeks 12 and 24, and BSA improvement from baseline at weeks 12 and 24. Assessments were performed at screening, at baseline, and every 4 weeks thereafter throughout the study. Safety of both treatment regimens was evaluated by recording the incidence of AEs (CTC version 2.0)^28^ and changes in laboratory assessments.

### Statistical analysis

The sample size of 240 patients per treatment arm was selected to detect a difference of ≥ 15% between treatment groups for the primary efficacy endpoint using a two-sided χ^2^ test at a significance level of 0·05 and 90% power. PASI 75 response for the monotherapy group at week 24 was assumed to be 50% based on results from a previous randomized phase III study using the intent-to-treat (ITT) analysis and nonresponder imputation (NRI) for missing data (Data on file, Amgen Inc., Thousand Oaks, CA, U.S.A.).

Efficacy analyses were performed using the ITT set (all randomized patients), and patients were analysed according to their original randomized group assignment. Missing postbaseline data were imputed using last observation carried forward for primary analyses of all efficacy endpoints. Sensitivity analyses were performed using NRI for missing data and observed cases for all efficacy endpoints. The safety evaluable set included all patients who had received ≥ 1 dose of study drug.

The primary analysis was performed after all week-24 data were finalized. The overall family-wise significance level for the analyses of primary and secondary efficacy endpoints was controlled at 0·05 using a combination of sequential testing and the Hommel procedure. The significance level for subgroup analyses was 0·05 without adjusting for multiplicity. For binary efficacy endpoints, between-group comparisons were conducted using the Cochran–Mantel–Haenszel (CMH) test with BMI and prior TNF-blocker exposure as the stratification factors. Comparisons for ordinal efficacy endpoints were conducted using the stratified CMH test and modified ridit scores because equal spacing between categories was not assumed. Between-group comparisons for continuous endpoints were made using the van Elteren stratified rank test adjusting for the stratification factors.

## Results

### Patients

**Fig 2:**
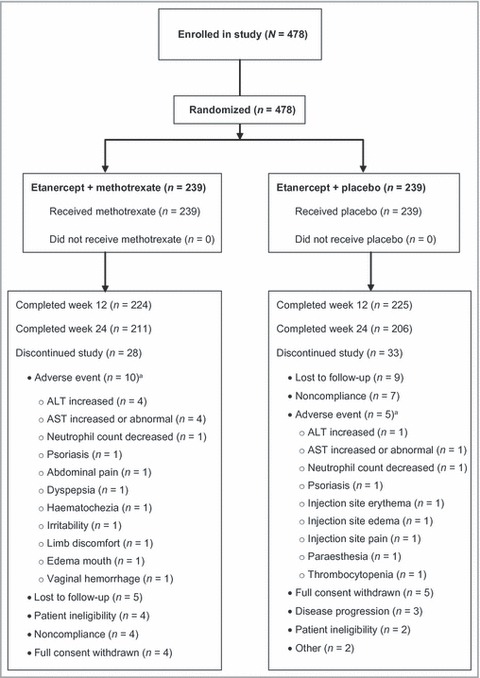
Patient disposition. ALT, alanine aminotransferase; AST, aspartate aminotransferase. ^a^Represents all adverse events leading to study withdrawal.

This study was initiated in November 2009, and the last patient completed follow-up in December 2010. All 478 patients enrolled in the study received ≥ 1 dose of study medication. Of the 239 patients in the combination group, 211 (88·3%) completed the 24-week study; in the monotherapy group, 206 of 239 patients (86·2%) completed the study (Fig. 2). The most common reasons for not completing the study were AEs (combination group, *n* = 10; monotherapy group, *n* = 5), loss to follow-up (*n* = 5; *n* = 9), noncompliance (*n* = 4; *n* = 7), and withdrawal of consent (*n* = 4; *n* = 5). One additional patient in the monotherapy group did not complete the study due to disease progression, which was also reported as an AE.

Patient demographics and baseline disease characteristics were generally well balanced across both treatment groups (Table 1). Most patients were men (66·9%) and white (74·5%), and the mean age was 44·1 years. The mean PASI score at baseline was 18·3, and mean psoriasis BSA involvement was 24·3%. In the combination and monotherapy groups, respectively, 220 (92·1%) and 217 (90·8%) had received previous psoriasis medication, and 145 (60·7%) and 128 (53·6%) had received prior systemic therapy or phototherapy. The mean duration of psoriasis at baseline was 17·4 years. One patient in the combination group (who completed the 24-week study) had stable moderate to severe plaque psoriasis for only 4·8 months (although disease duration of 6 months was an inclusion criterion).

**Table 1:** Baseline demographic and disease characteristics

Characteristic	Etanercept + placebo (*n* = 239)	Etanercept + methotrexate (*n* = 239)
Male sex, *n* (%)	167 (69·9)	153 (64·0)
Race, *n* (%)		
White	177 (74·1)	179 (74·9)
Black	10 (4·2)	4 (1·7)
Hispanic	39 (16·3)	42 (17·6)
Asian	9 (3·8)	10 (4·2)
Other	4 (1·7)	4 (1·7)
Age (years), mean (SD)	45·2 (12·8)	43·0 (13·1)
Median (Q1, Q3)	45·0 (36·0, 55·0)	43·0 (33·0, 52·0)
Weight (kg), mean (SD)	95·8 (24·5)	93·6 (24·2)
Median (Q1, Q3)	93·0 (79·6, 109·1)	89·0 (77·0, 104·0)
Height (cm), mean (SD)	172·0 (10·1)	170·8 (10·5)
Median (Q1, Q3)	172·7 (165·1, 177·8)	172·7 (164·0, 177·8)
BMI (kg m^−2^), mean (SD)	32·3 (7·5)	32·2 (8·1)
Median (Q1, Q3)	31·1 (27·1, 35·8)	30·9 (27·2, 35·6)
≤ 35 kg m^−2^, *n* (%)	173 (72·4)	172 (72·0)
> 35 kg m^−2^, *n* (%)	66 (27·6)	67 (28·0)
Psoriasis BSA affected, %, mean (SD)	24·2 (13·6)	24·4 (15·9)
Median (Q1, Q3)	20·5 (14·0, 30·0)	18·5 (13·8, 30·0)
PASI, mean (SD)	18·3 (6·6)	18·2 (8·2)
Median (Q1, Q3)	17·1 (13·3, 21·4)	15·9 (12·6, 21·9)
sPGA, *n* (%)		
Unknown	1 (0·4)	0 (0)
0	0 (0)	0 (0)
1	1 (0·4)	0 (0)
2	24 (10·0)	32 (13·4)
3	139 (58·2)	138 (57·7)
4	68 (28·5)	60 (25·1)
5	6 (2·5)	9 (3·8)
Duration of psoriasis, years, mean (SD)	16·9 (12·7)	17·9 (12·7)^a^
Median (Q1, Q3)	14·7 (6·0, 25·8)	15·8 (7·4, 26·0)
History of psoriatic arthritis, *n* (%)	50 (20·9)	58 (24·3)
Prior psoriasis therapy, *n* (%)	217 (90·8)	220 (92·1)
Prior systemic therapy, *n* (%)	100 (41·8)	108 (45·2)
Methotrexate	38 (15·9)	44 (18·4)
Oral retinoids	10 (4·2)	15 (6·3)
Steroids	10 (4·2)	14 (5·9)
Prior TNF-blocker therapy, *n* (%)	48 (20·1)	42 (17·6)
Etanercept	35 (14·6)	32 (13·4)
Adalimumab	13 (5·4)	10 (4·2)
Prior phototherapy, *n* (%)	66 (27·6)	84 (35·1)
UVB	55 (23·0)	66 (27·6)
PUVA	15 (6·3)	25 (10·5)
Prior topical nonsteroid use, *n* (%)	122 (51·0)	135 (56·5)
Vitamin D analogues	74 (31·0)	78 (32·6)
Tar compounds	66 (27·6)	66 (27·6)
Prior topical steroid use, *n* (%)	180 (75·3)	164 (68·6)
Other	123 (51·5)	120 (50·2)
Taclonex® (Leo Pharma Ballerup, Denmark): betamethasone+calcipotriene	86 (36·0)	72 (30·1)
Lotrisone® (Merck, Whitehouse Station,NJ, U.S.A.): betamethasone+clotrimazole	25 (10·5)	15 (6·3)

BMI, body mass index; BSA, body surface area; PASI, Psoriasis Area and Severity Index; Q1, first quartile; Q3, third quartile; sPGA, static Physician’s Global Assessment; TNF, tumour necrosis factor; UVB, ultraviolet B; PUVA, psoralen plus UVA.

a One patient had stable moderate to severe plaque psoriasis for 0·4 years.

The mean number of subcutaneous doses of etanercept that patients received during the study was 33·4 (maximum 36), and the mean number of oral doses of methotrexate/placebo was 21·8 (maximum 24). At week 24, 90·9% of patients in the combination group were receiving methotrexate 15 mg and 92·7% of patients in the monotherapy group were receiving placebo 15 mg. Three patients in the combination group received a methotrexate dose of > 15 mg weekly during the study, which was a protocol violation, including two patients at week 24. In the monotherapy group, 92·7% of patients were receiving placebo 15 mg at week 24; one patient was receiving placebo > 15 mg, which was a protocol violation. The mean duration of dosing was 151·9 and 152·5 days for subcutaneous and oral dosing, respectively.

### Efficacy

**Fig 3:**
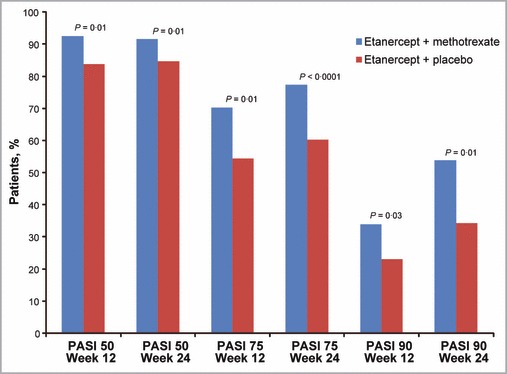
Proportion of patients with improvement in PASI of ≥ 50%, ≥ 75%, or ≥ 90% from baseline to weeks 12 and 24. PASI, Psoriasis Area and Severity Index.

**Fig 4:**
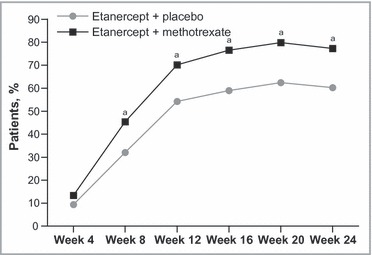
Proportion of patients with improvement in PASI of ≥ 75% at each measured time point from baseline to week 24. PASI, Psoriasis Area and Severity Index. ^a^*P* < 0·05, combination therapy vs. monotherapy.

At week 24, PASI 75 response (primary endpoint) was significantly higher for the combination group than the monotherapy group (77·3% vs. 60·3%; *P* < 0·0001) (Fig. 3). PASI 75 response at week 12 was also significantly higher in the combination group than the monotherapy group (70·2% vs. 54·3%; *P* = 0·01) (Fig. 3). After a reduction in the etanercept dose from 50 mg twice weekly (week 1–12) to 50 mg once weekly (week 13–24), PASI 75 response in both treatment groups increased or remained similar to those observed during the high-dose period (Fig. 4). Similar results occurred for PASI 50 at week 12 (combination, 92·4% vs. monotherapy, 83·8%; *P* = 0·01) and week 24 (91·6% vs. 84·6%; *P* = 0·01) and for PASI 90 at week 12 (34·0% vs. 23·1%; *P* = 0·03) and week 24 (53·8% vs. 34·2%; *P* = 0·01) (Fig. 3).

In patients with BMI ≤ 35 kg m^−2^ (72% of patients in each arm), between-group differences remained significantly in favour of the combination group across all PASI results at weeks 12 and 24 [e.g. PASI 75 results were 73·7% vs. 60·0% (*P* = 0·008) at week 12 and 78·9% vs. 64·1% (*P* = 0·003) at week 24], with the exception of PASI 90 at week 12 (36·3% vs. 27·6%; *P* = 0·10). In patients with BMI > 35 kg m^−2^ (28% of patients in each arm) responses were significantly higher in the combination group for PASI 75 and PASI 90 at weeks 12 and 24 but were similar for PASI 50 (data not shown). For example, PASI 75 responses were 61·2% vs. 39·1% at week 12 (*P* = 0·01) and 73·1% vs. 50·0% at week 24 (*P* = 0·007).

Among the patients who had received prior TNF-blocker therapy (Table 1), there was a trend for greater PASI responses in the combination group at weeks 12 and 24 (e.g. PASI 75 responses were 69·0% vs. 48·9% at week 12 and 76·2% vs. 57·4% at week 24), although between-group differences were only statistically significant for PASI 90 at week 24 (47·6% vs. 21·3%; *P* = 0·01). In patients without prior TNF-blocker therapy, significantly greater responses were seen in the combination group compared with the monotherapy for PASI 50, PASI 75 and PASI 90 at weeks 12 and 24 (data not shown). For example, PASI 75 responses were 70·4% vs. 55·6% at week 12 (*P* = 0·003) and 77·6% vs. 61·0% at week 24 (*P* = 0·0006).

**Fig 5:**
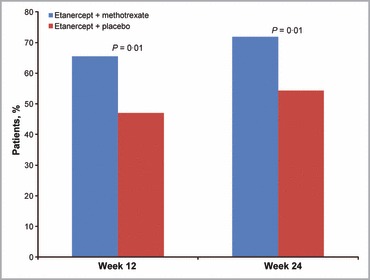
Proportion of patients with sPGA of clear (0) or almost clear (1) at weeks 12 and 24. sPGA, static Physician’s Global Assessment.

Significantly more patients in the combination group than the monotherapy group had sPGA of clear or almost clear at week 12 (65·5% vs. 47·0%; *P* = 0·01) and week 24 (71·8% vs. 54·3%; *P* = 0·01) (Fig. 5). Mean and median absolute BSA improvement from baseline to week 12 was numerically higher in the combination vs. monotherapy group (mean, 16·3% vs. 15·2%, combination vs. monotherapy, respectively, and median, 13·1% and 12·0%, respectively); at week 24, the values were 19·5% vs. 17·8% (median, 15·0% for both groups), respectively. Neither difference reached statistical significance. Mean BSA involvement at week 24 was 4·9% (median, 1·7%) in the combination group and 6·6% (median, 4·0%) in the monotherapy group [nominal (unadjusted) *P* < 0·0001].

### Safety

Overall, 74·9% of patients in the combination group experienced AEs compared with 59·8% in the monotherapy group (Table 2). Most AEs were mild or moderate in severity. The most common AEs in either group were nasopharyngitis, headache, upper respiratory tract infection and nausea. Infectious AEs were reported in 34·7% and 25·9% of patients in the combination and monotherapy groups, respectively. Six serious AEs (SAEs) occurred in five patients (combination group, *n* = 2; monotherapy, *n* = 3): lumbar spinal stenosis, synovial cyst and bacterial pneumonia in the combination therapy group; and asthma, cholecystitis and myocardial infarction in the monotherapy group. The incidence of elevated levels of hepatic transaminases considered AEs was higher in the combination therapy group compared with the monotherapy group [seven patients (2·9%) vs. four patients (1·7%)] (Table 2). Increased serum SGPT/ALT levels were observed in six (2·5%) patients in the combination therapy group and three (1·3%) patients in the monotherapy group. The corresponding values for increased/abnormal SGOT/AST levels were five (2·1%) and three (1·3%) patients. The percentages of patients with SGPT/ALT and SGOT/AST values CTC grade 1 (greater than the upper limit of normal) or above were higher in the combination therapy group (40·6% and 22·6%, respectively) than in the monotherapy group (25·5% and 12·1%, respectively). The percentages of patients with hepatic transaminase values CTC grade 2 (> 2·5–5·0 times the upper limit of normal; 2·9% and 2·1% vs. 1·3% and 2·5%) or above or grade 3 (> 5·0–20·0 times the upper limit of normal; 0% and 0·4% vs. 0·4% and 0%) or above were similar in the two treatment groups. Four patients in the combination therapy group and two patients in the monotherapy group withdrew due to increased transaminases (Table 2). No notable changes were observed in laboratory blood tests, and no haematological AEs were reported in ≥ 5% of patients.

**Table 2:** Adverse events occurring in patients in either treatment group through week 24

	Number (%) of patients	
	
	Etanercept + placebo (*n* = 239)	Etanercept + methotrexate (*n* = 239)
Any adverse event	143 (59·8)	179 (74·9)
Incidence ≥ 5%		
Nasopharyngitis	26 (10·9)	23 (9·6)
Headache	22 (9·2)	22 (9·2)
Upper respiratory tract infection	12 (5·0)	20 (8·4)
Nausea	7 (2·9)	13 (5·4)
Patients with any increased hepatic transaminases considered to be an adverse event^a^	4 (1·7)	7 (2·9)
SGPT/ALT increased	3 (1·3)	6 (2·5)
Leading to study withdrawal	1 (0·4)	4 (1·7)^b^
SGOT/AST increased/abnormal	3 (1·3)	5 (2·1)
Leading to study withdrawal	1 (0·4)	4 (1·7)^b^

SGPT/ALT, serum glutamic pyruvic transaminase/alanine aminotransferase; SGOT/AST, serum glutamic oxaloacetic transaminase/aspartate aminotransferase.

a Includes increased SGPT/ALT, increased SGOT/AST, increased hepatic enzymes, abnormal liver function test, and/or abnormal SGOT/AST considered to be adverse events. ^b^Represents the same four patients.

Withdrawals due to AEs were infrequent in both groups [combination, *n* = 10 (4·2%); monotherapy, *n* = 6 (2·5%)], and none of the AEs leading to withdrawal was considered to be serious or infectious. No specific AEs leading to withdrawal occurred in > 1 patient per treatment group, with the exception of increases in hepatic transaminases, which caused more withdrawals in the combination group (Table 2). The increases in hepatic transaminases were not considered serious in any patient, and all events were mild or moderate in severity.

Two patients in the combination group reported incidences of cutaneous squamous cell carcinoma during the study (two incidences in one patient, and one incidence in the other patient). None of these malignancies was considered serious, and both patients completed the study. Injection site reactions were reported in 15 patients (6·3%) in the combination group and 18 (7·5%) in the monotherapy group; all of these AEs were mild or moderate. No opportunistic infections or deaths occurred during the study.

## Discussion

Combination therapy with etanercept plus methotrexate has been shown to be efficacious and has acceptable tolerability in patients with RA.^21^–^25^ Etanercept plus methotrexate combination therapy is approved in the U.S.A.^8^ for use in patients with psoriatic arthritis who do not respond adequately to methotrexate alone, and the European League Against Rheumatism recommends the combination of a synthetic disease-modifying antirheumatic drug (DMARD) (such as methotrexate) plus a TNF blocker in patients with psoriatic arthritis with active arthritis and an inadequate DMARD response,^29^ yet this therapeutic combination has not been widely investigated in psoriasis.^26^,^27^ This study evaluated etanercept monotherapy vs. etanercept plus methotrexate in patients with moderate to severe plaque psoriasis. It is important to note that combination therapy with etanercept plus methotrexate has not been approved for use in patients with psoriasis.

Etanercept plus methotrexate was more effective than etanercept monotherapy as measured by a significantly higher PASI 75 at week 24 (primary endpoint) and a significantly higher PASI 75 at week 12, PASI 50 at weeks 12 and 24, and PASI 90 at weeks 12 and 24. Additionally, the proportion of patients with an sPGA of clear or almost clear was significantly higher for the combination arm than the monotherapy arm at weeks 12 and 24. Improvement in BSA score from baseline to weeks 12 and 24 was numerically higher for the combination arm, with both treatment arms achieving low mean BSA scores at week 24. In general, efficacy results remained similar or increased after the etanercept dose was decreased from 50 mg twice weekly (weeks 1–12) to 50 mg once weekly (weeks 13–24), regardless of treatment group.

Despite being associated with dose-related AEs such as hepatotoxicity and myelosuppression, methotrexate is widely used and is generally effective as monotherapy and in combination with other therapies (including biologics) in patients with psoriasis.^7^,^30^–^34^ The results from the combination arm in the present study are similar to those from an open-label pilot study of etanercept plus continuous methotrexate (*n* = 31) vs. etanercept plus tapered/discontinued methotrexate (*n* = 28) in patients with psoriasis.^27^ In that earlier trial,^27^ patients in the continuous methotrexate arm had significantly better outcomes in terms of sPGA and PASI 75, with a similar safety profile to patients in the tapered methotrexate arm.

The safety and tolerability profiles for both the combination and monotherapy groups were acceptable in this trial. More patients in the combination arm than in the monotherapy arm experienced at least 1 AE (74·9% vs. 59·8%), but most AEs were mild or moderate in severity. In general, the types and incidences of AEs reported here are consistent with those seen in earlier psoriasis trials with etanercept monotherapy^11^,^13^,^14^ or methotrexate monotherapy,^19^,^20^,^35^ as well as in the previous small combination therapy trial of etanercept plus methotrexate.^27^ The safety profile of the combination regimen is also consistent with previous randomized combination trials with etanercept and methotrexate in RA.^21^–^24^,^36^ Study withdrawals because of AEs were infrequent and generally were single occurrences in each treatment group. The incidence of and withdrawals due to elevated levels of hepatic transaminases was higher in the combination therapy group than in the monotherapy group. Although increased liver enzyme levels are associated with methotrexate treatment,^20^,^37^ whether the occurrences in our study are related solely to methotrexate or to an additive effect of etanercept is not known. Most AEs and SAEs were noninfectious in nature, and no clinically significant haematological abnormalities, opportunistic infections, or deaths occurred during the study.

Previous psoriasis studies have shown that biologic therapy can be used effectively in combination with other systemic or topical antipsoriatic therapies.^38^–^41^ Alefacept plus other psoriasis treatments, including methotrexate, appears to be efficacious and well tolerated in patients with chronic plaque psoriasis.^38^,^39^,^41^ In a retrospective long-term cohort study, 70% of patients receiving infliximab plus either methotrexate or azathioprine had PASI 75 responses at week 14, and 15 of 23 patients continued to receive treatment for > 1 year.^40^

The principal limitations of our study are the lack of a methotrexate monotherapy group and the 24-week study period. Although the combination of etanercept plus methotrexate demonstrated superior efficacy to etanercept monotherapy in this study, evaluation in a longer study would more firmly establish the efficacy and safety of the combination regimen. Long-term clinical efficacy and safety of etanercept alone have been reported for up to 2·5 years in open-label studies of patients with moderate to severe psoriasis,^42^,^43^ and long-term data (up to 2 years) demonstrate the efficacy and safety of the etanercept and methotrexate combination in patients with early, active RA.^44^

In conclusion, adding methotrexate to etanercept resulted in increased efficacy with acceptable tolerability and safety compared with etanercept alone in adults with stable moderate to severe plaque psoriasis who were candidates for systemic therapy or phototherapy.

**What’s already known about this topic?**

- Despite demonstrated efficacy and tolerability in rheumatoid arthritis, etanercept in combination with methotrexate has not been widely evaluated in patients with psoriasis.

**What does this study add?**

- In adults with moderate to severe plaque psoriasis who were candidates for systemic therapy, etanercept plus methotrexate demonstrated increased efficacy over etanercept alone with an acceptable increase in non-serious adverse events.
- Etanercept plus methotrexate may be a viable regimen in patients with psoriasis.

## Appendix

*Conflicts of interest statements*: A.B.G. is a consultant and/or advisory board member for Abbott, Actelion, Amgen, Astellas, Beiersdorf, Bristol-Myers Squibb, Can-Fite, Celgene, Centocor (Janssen), Dermipsor, Incyte, Lilly, Merck, Novartis, Novo Nordisk, Pfizer, TEVA, and UCB and is a recipient of research/educational grants paid to Tufts Medical Center by Abbott, Amgen, Celgene, Centocor (Janssen), Immune Control, Novartis, Novo Nordisk, Pfizer, and UCB. R.G.L. has served as an investigator, on the scientific advisory board, and speaker for Abbott, Amgen, Centocor, and Pfizer, and as an advisor and investigator for Celgene, Novartis, and Johnson & Johnson. B.E.S. has served as an advisor, consultant, investigator, and speaker for Abbott, Amgen, and Centocor, and as an advisor, consultant, and investigator for Celgene, Novartis, Maruho, and Pfizer. K.A.P. has been a consultant, advisory board member, and investigator for Abbott, Amgen, Celgene, Centocor, Janssen-Ortho, MedImmune, Merck, Pfizer, Schering-Plough, and Wyeth (Wyeth was acquired by Pfizer in October 2009); has consulted for Astellas and UCB; and has served as a speaker for Abbott, Amgen, Celgene, Janssen-Ortho, Pfizer, Schering-Plough, and Wyeth. P.K., K.C., E.H.Z.T., M.H., and G.K. are employees and stockholders of Amgen Inc.
